# Information and Communication Technologies to Support Early Screening of Autism Spectrum Disorder: A Systematic Review

**DOI:** 10.3390/children8020093

**Published:** 2021-02-01

**Authors:** Lorenzo Desideri, Patricia Pérez-Fuster, Gerardo Herrera

**Affiliations:** 1 AIAS Bologna Onlus, 40134 Bologna, Italy; 2 Autism and Technologies Laboratory, University Research Institute on Robotics and Information and Communication Technologies (IRTIC), Universitat de València, 46010 València, Spain; patricia.perez-fuster@uv.es (P.P.-F.); gerardo.herrera@uv.es (G.H.)

**Keywords:** autism spectrum disorder, screening, information technology, primary care

## Abstract

The aim of this systematic review is to identify recent digital technologies used to detect early signs of autism spectrum disorder (ASD) in preschool children (i.e., up to six years of age). A systematic literature search was performed for English language articles and conference papers indexed in Pubmed, PsycInfo, ERIC, CINAHL, WoS, IEEE, and ACM digital libraries up until January 2020. A follow-up search was conducted to cover the literature published until December 2020 for the usefulness and interest in this area of research during the Covid-19 emergency. In total, 2427 articles were initially retrieved from databases search. Additional 481 articles were retrieved from follow-up search. Finally, 28 articles met the inclusion criteria and were included in the review. The studies included involved four main interface modalities: Natural User Interface (e.g., eye trackers), PC or mobile, Wearable, and Robotics. Most of the papers included (n = 20) involved the use of Level 1 screening tools. Notwithstanding the variability of the solutions identified, psychometric information points to considering available technologies as promising supports in clinical practice to detect early sign of ASD in young children. Further research is needed to understand the acceptability and increase use rates of technology-based screenings in clinical settings.

## 1. Introduction

Autism spectrum disorder (ASD) is a category of neurodevelopmental disorder characterized by persistent deficits in social communication and social interaction across multiple contexts as well as restricted, repetitive patterns of behavior, interests, or activities [1]. The care and social needs of preschool children with ASD (typically up to six years of age), in particular, are significant [2,3], usually extend to parents and siblings [2,4,5], and require substantial community resources [2,6,7]. In response to these needs, early detection of ASD has become a priority for primary care and other community settings [8] to provide early intervention services and to improve outcomes [2,9]. 

Timely (i.e., early) identification of ASD may be achieved by implementing screening methods and instruments that allow health and other professionals (e.g., social care, educators) for a rapid and relatively inexpensive evaluation of this condition in young children [10]. Screening measures that are suitable for use to identify ASD are already available and can vary by format (e.g., parent-report versus direct observation), scope, and target population [11]. With regard to the scope of the screening instruments, “broadband” screens cover multiple developmental domains, while “narrow” screens cover only those signs and symptoms specific to the condition of interest [11,12]. With regard to the target population, screening instruments can be used to conduct universal population-wide testing (also referred to as “universal screening” or Level 1 screening), or to identify possible signs of ASD in high-risk populations, such as siblings of children with ASD or those referred for speech or other developmental concerns to community pediatric services (also referred to as Level 2 screening) [12,13]. 

A number of relevant systematic reviews have examined the use of screening instruments for the identification of ASD in pediatric populations (o-6 years; see [13,14] for an overview of recent systematic reviews). Current evidence suggests that the most used and reliable instruments available to clinicians (e.g., pediatricians; developmental/child psychologists, child psychiatrists) are in the form of questionnaires, checklists, or observation scales where parents or clinicians are required to report/observe overt behavioral signs of ASD (e.g., limited smiles, eye contact) [11]. Advantages of these approaches have been extensively recognized and include high predictive values, ease of use, speed of administration, and limited or no specific administration/scoring training [13,14]. 

Notwithstanding the advantages, and the widespread implementation of these instruments in primary and community care settings as well as specialized services [15], screening instruments are still underused in routine clinical practice because of a number of challenges, such as lack of time, disruption of workflow, lack of familiarity with screening tools, difficulty with scoring, as well as lack of office-based systems for making referrals and monitoring outcomes (for an overview see [9]). As a consequence of these challenges, in spite of the possibility to reliably diagnose ASD in children during the first two years of life [2,12,16,17], current evidence reports that the diagnosis remains delayed in many children [18,19,20]. For instance, in a recent survey involving 1223 families and 760 professionals in 14 European countries [18], only 3.1% of the parents reported having noticed problems after responding to a specific ASD screening survey. In addition, the average age at diagnosis was 36.4 (SD = 17.7) months, with most diagnoses occurring between 32 and 46 months. In light of this evidence, it has been suggested that more effective screening strategies are needed to reduce the proportion of children who receive a late diagnosis or remain undetected [14,21,22]. Specifically, screening strategies are needed that (a) are able to reduce the workload of clinicians, (b) can be easily implemented within routine clinical practice, and (c) are psychometrically sound. 

Over the past decade, advances in information and communication technologies (ICT) have opened innovative and promising scenarios for clinicians to improve both identification, treatment and support (e.g., [23,24]) of children with ASD. Such solutions may be further used to help clinicians (and other stakeholders) improve early screening of ASD in that they may allow them monitoring young children’s behaviors in clinical settings as well as in their natural environments [25]. 

This paper is aimed at providing a picture of the different technology-based solutions to screen for ASD reported in the literature since 2010. This starting date was chosen as it represents the time period when most of the current mobile devices (e.g., touch-screen devices) were first introduced in the market [26]. For the scopes of the present study, we use the term “technology” to refer to any ICT-based product, either mainstream (e.g., smartphone, tablet) or emergent (e.g., robots), that was tested for the purpose of screening for ASD. 

Accordingly, our objectives are to review studies that implemented technological solutions specifically developed to screen for ASD in clinical practice, laboratory settings, at children’s homes, or in community settings, and to determine the level of development (maturity) reached by those solutions, as well as their expected contribution in supporting ASD screening practices. This review focuses on both Level 1 and Level 2 screeners. While Level 1 screening tools may be used to identify children at risk of ASD in the general population, Level 2 screeners are mainly used to distinguish between children with signs of ASD and those with other developmental concerns (e.g., language disorders, intellectual disability, other neurodevelopmental disorders). In this view, screening for ASD may be conceived as a multistep process, according to which children who fail a Level 1 screening would require a secondary (i.e., Level 2) screener before being referred to a more comprehensive and diagnostic assessment process [12,13,27] Providing such a comprehensive overview of the literature (including both levels of ASD screening) was thought to be useful to guide researchers and professionals in their choice of technology options in daily practice, as well as to stimulate their research initiatives aimed at adding essential evidence about technology-based ASD screeners. 

## 2. Materials and Methods

### 2.1. Search Strategy

A systematic search was conducted following the Preferred Reporting Items for Systematic Reviews and Meta-Analyses (PRISMA) reporting guideline recommendations [28] to identify studies reporting on commercially available ICT solutions or assistive technology products to screening children aged 0–6 years for ASD. The search was performed using the following academic databases: MEDLINE, consulted through the free electronic access PubMed; PsycINFO, ERIC, and CINAHL consulted through EBSCOHost; and Web of Science. IEEE and ACM digital libraries were also included. Search terms related to children, ASD, information technology and screening were used, and the search queries conducted with each database are listed in Appendix A. 

**Figure 1:**
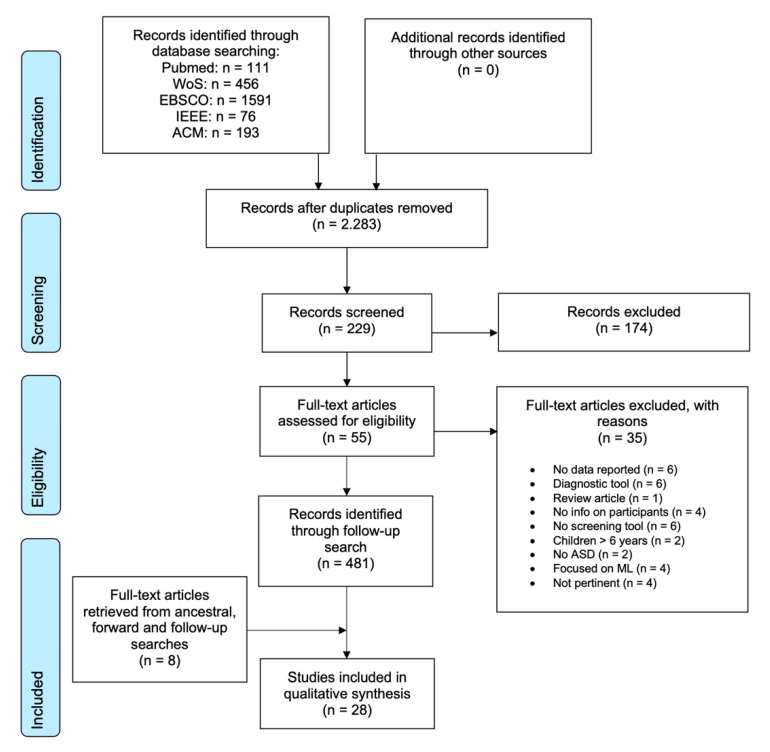
PRISMA flowchart of the articles’ selection process.

The search was conducted by the first author in February 2020 and was restricted to English-language, peer-reviewed journals, and conference papers published as of January 2020. Figure 1 illustrates the search process and outcome. Initially, 2427 article titles were identified. The titles were reduced to 2283 once the duplicates and articles not in English were removed. The three authors assessed the eligibility of titles and abstracts. If the title of an article matched pre-specified inclusion and exclusion criteria (see Appendix B), then the abstract was further read by all raters. Full texts were downloaded to judge the article’s eligibility for the review if the abstract matched further specified inclusion and exclusion criteria (details in Appendix B). 

On this basis, 55 full-text articles were downloaded and fully read by the first author, who finally selected 20 of them according to specific inclusion/exclusion criteria (see below). Subsequently, an ancestral and forward search (i.e., Google Scholar’s “cited by” function) was conducted by the first author using the 55 articles originally reviewed. In addition, in order to keep up with the rapid publication rate in ASD research, as well as to identify research in this area during the Covid-19 emergency, a follow-up search was conducted on Google Scholar (using the search terms “autism” and “screening”) to identify papers published between March and December 2020. The Google Scholar search yielded in total 481 titles, of which four were included in the review. The additional forward and ancestral searches led to the finding of further four papers and so 28 articles were finally included in the review.

### 2.2. Full-Texts’ Inclusion and Exclusion Criteria

The following inclusion criteria were used in selecting the studies for the review:

1. The paper had to report on the development and/or implementation of technology arrangements (whether they are commercially available or not, independently if they have been specially developed for screening or adapted from solutions available for different purposes) aimed at detecting early signs of ASD across a range of clinical (e.g., primary care; specialized clinics/services), and other settings such as laboratory, home, or school.
2. The studies had to target children aged ≤6 years. Studies involving broader age ranges were included providing that they involved children within the aforementioned age group (i.e., age ≤ 6 years).
3. The studies had to provide quantitative information on the capability of the technology (or the technology-based approach) of:
  - Screening for ASD at the population level (Level 1 screening; L1), such as children evaluated by primary care physicians, or
  - Screening for ASD in a subsample of the population identified as at risk for the disorder (Level 2 screening; L2), such as a referred clinical sample with a variety of developmental concerns, siblings of children with ASD, pre-term children, children with genetic syndromes that are usually associated with ASD, or children with a diagnosis of other neurodevelopmental disorders [29].

Excluded from the review were studies:

1. Reporting on a retrospective analysis of existing databases of evaluation records which were not directly implemented in the aforementioned applied settings and/or did not involve the target users (i.e., health professionals; caregivers);
2. Focusing on invasive or non-invasive techniques to investigate biological processes and structures (e.g., electroencephalography, brain imaging, electrodermal activity);
3. Using technology to investigate physiological (e.g., heart rate; eye movements), behavioral (e.g., vocal or movement patterns; crying), or cognitive differences between children with/at risk of ASD and controls not for the purpose of developing a screening tool;
4. Providing training to professionals on the use of a screening tool.

### 2.3. Data Coding and Extraction

The studies that met the aforementioned inclusion criteria were coded in terms of participant characteristics (i.e., number, age-range and sex), target users of the technology, indicators used to assess ASD condition, types of technology used, context(s) of use of the technology, screening level, and maturity of the technology. A brief description of each technology identified, the methodology for its evaluation, and its psychometric properties were also provided.

Country of origin of the study was reported based on (i) the information provided in the methodology, or (ii) the affiliation of the corresponding or the first author of the paper. To classify the types of technologies used in each paper, we adapted the classification proposed by Kientz et al. [30] which includes six different types of interface, namely (a) Personal computers (PC) or mobile, (b) shared interactive interfaces, (c), virtual, augmented, and mixed reality, (d) sensor-based and wearable, (e) natural user interfaces, and (f) robotics. Likewise, to rate the maturity of the technology identified, we used the maturity levels proposed by Kientz et al. [30], that is, (a) functional prototype or (b) publicly available. Specifically, a functional prototype refers to technology that has been developed and interacted with the intended users for the target purposes but may require assistance with setup, use, or maintenance. Technologies classified as publicly available, in contrast, refer to commercial products, software that is open source, or applications available for download on websites or on mobile marketplaces (even if no longer available at the time of the present review).

When not specifically mentioned in the paper, we conceived L1 screening as applying to (a) all children regardless of the risk status (such as the M-CHAT), (b) tools implemented to assess children during routine pediatric visits, (c) experimental or observational studies that compared children with a diagnosis of ASD with neurotypical children. In contrast, we conceived L2 screening tools as (a) targeted at children already identified as being at increased risk (e.g., due to a positive family history), and/or (b) used to distinguish between ASD and other neurodevelopmental disorders.

Finally, we extracted relevant information on psychometric properties typically used for screeners, when available. Metrics extracted included (1) sensitivity (the percent of cases with ASD classified by the instrument as ASD); (2) specificity (the percent of cases without ASD classified as not having ASD); (3) positive predictive validity (the percent of cases accurately predicted as having ASD); and (4) negative predictive validity (the percent of cases accurately predicted as not having ASD). Measures of accuracy in distinguishing between clinical and non-clinical groups were also considered relevant.

### 2.4. Inter-Rater Agreement

The first author calculated the inter-rater agreement between the three raters pairwise on all titles (n = 2283) and abstracts (n = 229). Based on rating criteria (see details in Appendix B), proportional agreement on the titles and abstracts was calculated by taking the number of agreements and dividing this by the number of agreements plus disagreements, multiplied by 100. Their agreement ranged between 65% and 84% for the titles, and 93% and 96% for the abstracts.

Consensus was reached on the titles and abstracts with disagreement after the three raters reviewed them again together. Inter-rater agreement was also checked on the summary points of the variables coded (see above). The first author extracted the information for the 28 papers included and a second rater extracted the information for eight randomly selected papers. The two authors agreed on 149 of the 152 summary points checked (i.e., 19 summary points per article multiplied by 8 articles). Following the same formula used above, the percentage of agreement was 98%. The two raters then discussed the discrepancies until a 100% agreement was reached.

## 3. Results

### 3.1. Overview of the Results

We identified 28 studies that used mainstream or adapted information technologies to screen children up to 6 years for ASD (see Table 1). Seven of the included studies [22,31,32,33,34,35,36] involved children recruited from primary care or pediatric services, while five studies involved children referred to tertiary care or specialized ASD centers [37,38,39,40,41]. A total of 7308 children participated in the studies. Of these, 3498 were males, 1851 females. In nine studies gender information were missing. 

Ages of the children involved in the studies varied greatly. Two studies involved children from 6 to 18 months [42,43]. Six studies involved children within the 10 to 48 months range [36,40,41,44,45,46], seven studies involved participants aged between 16 and 30 months [22,25,31,32,34,35,47], and four studies involved children within the 18- and 72-months range [37,39,48,49]. Three studies involved a sample of children aged between 48 and 72 months [33,50,51]. The remaining six studies involved samples within age ranges that included children with ASD both up to six years and older [38,52,53,54,55,56]. 

The majority of the studies reported in the papers identified was conducted in the USA (n = 19). Seven studies were conducted in as many countries, including China [51], Peru [49], UK [48], Italy [52], France [54], Colombia [56], and Sri-Lanka [46]. Two papers either did not provide information [55] or provided unclear information as to the country of origin of the participants recruited [45].

### 3.2. Types of Technologies Used

**Figure 2:**
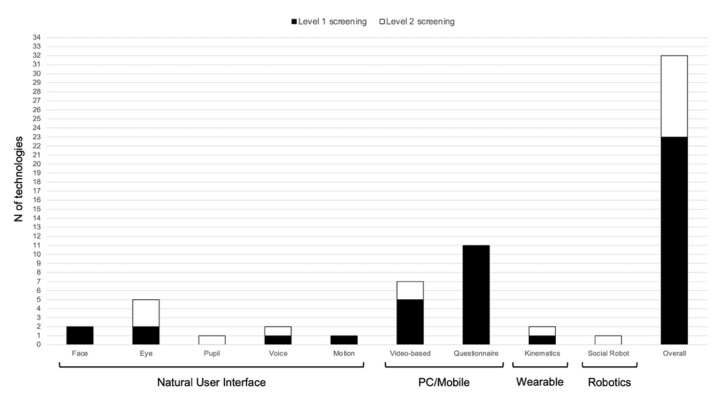
Frequency of technologies used in the papers included grouped according to interface category.

The studies included in the review involved four main interface modalities, namely (a) natural user interface (NUI), (b) PC or mobile, (c) wearable, and (d) robotics. Figure 2 illustrates the frequencies of the different interfaces used within each category. 

The former category (i.e., NUI) included 11 papers. Of these, five papers involved the use of eye trackers [38,40,41,49,51], two studies used voice-based recording systems [44,53], two studies employed face-recognition to detect facial expressions [25,36], one paper involved motion recognition using touch screen sensor technologies [48], and one paper tracked pupil diameter [54]. 

The second category (i.e., PC or mobile) included 16 papers. The studies reported by Abbas et al. [37] and Kanne et al. [39] were included in both categories (i.e., PC and Mobile) as they combined the two strategies within the same application. In a similar vein, the studies reported by Egger et al. [25] and Carpenter et al. [36] were included both in the NUI and PC/Mobile category. Accordingly, 11 papers reported on the use of computerized solutions (PC or mobile platforms) to administer parent-reported questionnaires [22,31,32,33,34,35,37,39,46,47,55], and seven papers employed screening tools in which videos were collected from [37,39,43,50] or showed via [25,36,42] parents’ mobile/PC devices. 

The third category (i.e., wearable) included two papers [45,52] that used wearable sensors to track the kinematics of children’s movements while they were performing specific reaching and grasping movements.

The fourth category (i.e., robot) included one paper [56] that reported on the use of a humanoid robot to assess joint attention skills.

### 3.3. Screening Level

The majority of the papers included in the review (71%; n = 20) involved the use of L1 screening tools. A detailed analysis of the differences between the two screening approaches according to relevant study characteristics (e.g., target population; type of interface used) was not performed because of the relatively low number of L2 papers. However, it should be noted that all papers involving parent-reported questionnaires (n = 11) focused on L1 screening approach. In contrast, papers involving L2 screening tools were mostly focused on using objective screening measures such as eye-tracking (n = 3), audio recording (n = 1), or kinematics (n = 1). The identified papers were grouped according to the different age ranges of the populations involved. Detailed descriptions of each study are provided in Table 2.

#### 3.3.1. L1 Screening Tools

##### Solutions Tested with Children up to 30 Months

Nine papers were identified that involved children in the 16–30 months age range [22,25,31,32,34,35,36,46,47]. Of these, two papers reported on studies aimed at adapting the M-CHAT for its administration via tablet [3,31]. Benefits of the use of tablet over the traditional paper-and-pencil form have been clearly highlighted by Campbell et al. [31], who documented that after implementation of the digital M-CHAT (a) the proportion of children screening positive with accurate documentation in the Electronic Health Records (EHR) increased from a mean of 54% to 92%, and (b) the proportion of physicians referring a child for a developmental assessment after a positive score increased from 56% to 100% (see also Major et al. [58] for secondary analyses). 

Three studies reported on the use of automated EHR [22,32,35] to facilitate screening procedures within pediatric clinics. Both Bauer et al. [22] and Downs et al. [32] (see also [59], not included in this review) implemented the Child Health Improvement Through Computer Automation system (CHICA). CHICA is a computer decision support system developed to facilitate surveillance and screening for ASD in primary pediatric care services by implementing automated administration and scoring of the M-CHAT. Although encouraging results were observed in terms of increased screening of children for ASD, in both studies concerns were raised about the physicians’ response to the alerts that a patient had a concerning M-CHAT. In a similar line of investigation, Schrader et al. [35] implemented the Smart Early Screening for Autism and Communication Disorders (Smart ESAC) in a pediatric service. Results indicated a statistically significant reduction in the average age of referral after the implementation of the Smart ESAC compared to the 16 years prior to system implementation.

Ben-Sasson et al. [47] created a survey through which parents recruited via online advertisement could describe in their own words their concerns regarding their child’s social-communication development. Parents were further asked to complete the M-CHAT-R/F and the Autism Spectrum Quotient (ASQ) questionnaire. The authors were able to reliably predict the risk status of a child being on the spectrum by supplementing their written descriptions with only one of 11 questions taken from the M-CHAT-R.

Wingfield et al. [46] developed a mobile-based questionnaire with automatic scoring to be administered by non-specialist health/social workers in low-income countries. The system is a set of 21 “yes-no” questions for the parents. Preliminary evidence shows high accuracy in distinguishing between already diagnosed children with ASD and their neurotypical peers.

Finally, two studies used mobile devices to track facial expressions [25,36]. Egger et al. [25] developed an iPhone/iPad-based application to screen for signs of ASD in the general population. The app includes a short set of questionnaires as well as four brief videos. While the child watches the videos, the camera embedded on the device records his or her face. The recorded videos are thus uploaded by the caregivers on a server that automatically analyzes the child’s facial expressions and attention to estimate the risk of ASD. Preliminary results indicated that (a) the majority of parents were willing to upload the full videos of their children; and (b) significant associations were found between emotions and attention and age, sex, and autism risk status (based on the M-CHAT scores). Similar encouraging results were reported by Carpenter et al. [36] who seemingly used the same system as that tested by Egger et al. [25].

##### Solutions Tested with Children up to Six Years

Vargas-Cuentas et al. [49] presented a 1-min video displaying a social scene with playing children and an abstract scene with moving shapes on either side of the screen. Observer’s eye gaze while watching the videos were automatically tracked to assess spatial preference. Results from the proof-of-concept study comparing the eye gaze of children with ASD over those of their neurotypical peers as controls showed that the former group spent 26.9% to 32.8% of the time gazing at the social scene, compared to 44.2% to 50.7 of the control group.

Anzulewicz et al. [48] used two commercially available gameplays running on iPad to record children’s movements while interacting with the device. Differences between children with a diagnosis of ASD and their neurotypical peers were estimated by means of a machine learning algorithm which resulted highly accurate in distinguishing the two groups based on the sole kinematics information.

Wan et al. [51] used an eye tracker to distinguish children with ASD from their neurotypical peers. They developed a rapid screening session which involved the presentation of a video showing a speaking girl for a very brief time interval (i.e., about 10 s). Automatic analysis of children’s gaze produced reliable results in distinguishing between the two groups (i.e., ASD and neurotypical). Despite several differences in gazing behavior between the two groups while watching the speaking face, only the fixation times at the moving mouth and body could significantly discriminate the ASD group from the control group with acceptable classification accuracy. 

Duda et al. [33] tested the Mobile Autism Risk Assessment (MARA) screening tool with children aged between 16 months and 17 years referred to a developmental-behavioral pediatric clinic. MARA is a 7-item parent questionnaire that can be administered via an electronic platform with automatic scoring. Before its implementation in a clinical setting, the questionnaire was validated in a series of preliminary studies [60]. Results from the implementation study showed that children who received a clinical ASD diagnosis were more likely than those without a clinical ASD diagnosis to receive a MARA score that was indicative of ASD. Importantly, the respondent could complete the MARA questionnaire either at home or in the clinic. Based on this preliminary clinical validation, two further papers by Abbas et al. [37] and Kanne et al. [39] tested the Cognoa application involving children aged between 18 to 72 months. Cognoa is a mobile-based application (i.e., tablet; smartphone) using the same algorithm used in MARA. It follows a two-stage approach to ASD screening whereby a parent (a) answers to a 15-item questionnaire and (b) uploads through the mobile phone at least 1–2 min. videos of the child being rated recorded in different everyday scenarios (e.g., mealtime, playtime, or conversations). Videos are then rated by specialized assessors to determine the need for further assessment. Results indicated that the Cognoa (a) performed similarly to other screening measures (i.e., MCHAT-R/F; SCQ; SRS; CBCL-ASP), and (b) was able to reliably screen all children in the 18–72-month age range, thus covering the screening age gap between 30 months and 48 months.

In a similar vein, Tariq et al. [50] created a mobile web portal to test the ability of machine learning to reliably detect autism based on short home videos of children. The results suggest that machine learning may enable rapid ASD detection outside of clinics, thus reducing waiting periods for access to care and reach underserved populations. 

#### 3.3.2. L2 Screening Tools

##### Solutions Tested with Children up to 18 Months

Two papers were included that involved children up to 18 months [42,43]. Young et al. [42] developed a web-based application named Video-referenced Infant Rating System for Autism (VIRSA). The application is intended to be used by parents and shows pairs of videos of parents and infants playing together. After the presentation of each pair of videos, the respondent is asked to make judgments of which video is most similar to the child being rated. The application was tested involving infants with an older sibling with ASD, with preliminary results showing that VIRSA could correctly identify all children diagnosed with ASD at 18 months.

Talbott et al. [43] reported on the feasibility of instructing parents to administer specific semi-structured behavioral probes using the Telehealth Evaluation of Development for Infants (TEDI). This approach resulted reliable and acceptable to parents, although the sample involved was relatively small (i.e., 11 children).

##### Solutions Tested with Children up to 48 Months

Four papers were identified involving children aged between 10 and 48 months. Pierce et al. [41] developed the GeoPref test based on the assumption for which preference for geometric shapes over social content might be a reliable biomarker of ASD (see also [61]). The test involved the use of an eye-tracker that monitored the gaze behavior of the child while he or she was watching a video representing dynamic geometric images paired with a video representing dynamic social images. Results showed that a subset of ASD toddlers who fixated on the geometric images >69% of the time was accurately identified as being on the spectrum with high specificity. These promising results were further replicated by Moore et al. [40] using longer and more complex social scenes (see also [62] for the use of the GeoPref test as a symptom severity prognostic tool).

Wedyan and Al-Jumaily [45] conducted a proof-of-concept study to investigate the use of a wrist-worn light sensor to monitor object manipulation skills of children while they inserted a ball into a plastic tube. Automatic classification of the movement data was able to differentiate children at high risk of ASD from those at low risk with high accuracy. 

Oller et al. [44] used the Language ENvironment Analysis (LENA) system to collect whole day audio recordings of infants in their homes. They further developed an automated approach to data analysis that was able to differentiate between vocalizations produced by neurotypical children from those produced by children with ASD or language delay.

##### Solutions Tested with Children up to Six Years and Older

Two papers were included in this group. Frazier et al. [38] estimated an Autism Risk Index by means of eye-tracking technology used to record fixations of children while presented with a variety of social and nonsocial visual stimuli. The results indicated that, for children with ASD up to 48 months and older, the index was able to classify their clinical condition with very good accuracy. Classification accuracy was also strong for children aged 30 months or younger. 

Ramirez-Duque [56] tested the feasibility of using a social robot with a humanoid appearance to elicit and assess joint attention in children with a diagnosis of ASD. The robot was used in triadic interactions. The results showed that children with ASD produced less joint attention-related behaviors compared to a control group of children with other neurodevelopmental disorders. 

### 3.4. Technology Maturity

**Figure 3:**
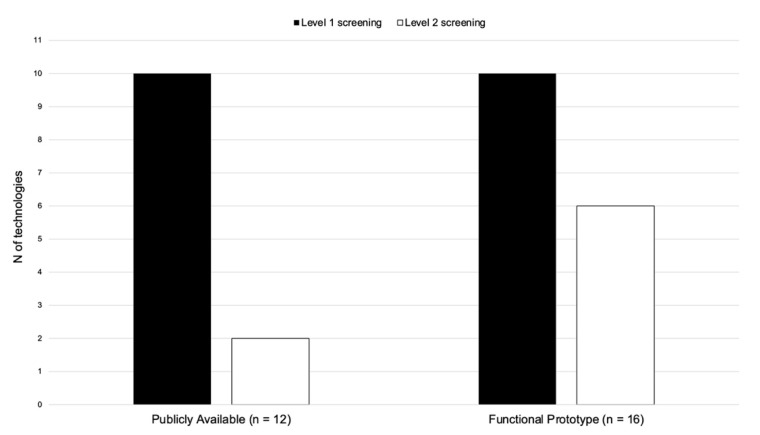
Papers included in the review grouped according to maturity and screening levels.

About half (57%; n = 16) of the papers identified reported on the use of the screening tools were classified as reporting on a Functional Prototype (see Figure 3). Of these prototypes, 10 (62%) were L1 screening tools. Similarly, of the papers reporting on technologies classified as publicly available (n = 12), the majority (92%; n = 11) reported on L1 screening tools. Almost all the screening tools classified as publicly available (n = 10) were PC/Mobile interfaces used to administer parent-reported questionnaires for L1 screening. In contrast, functional prototypes were mostly represented by NUI interfaces (56%; n = 9), of which five involved the use of eye trackers.

### 3.5. Psychometric Properties

Table 2 reports key information on the psychometric properties of the screening tools assessed in the papers identified. Five studies reported all the four metrics considered relevant for a screening tool (i.e., Sp; Se; PPV; NPV), and 18 papers reported at least one of such psychometric metrics or provided information of accuracy in detecting risk of ASD. Of the papers reporting psychometric information (n = 23), eight papers reported sensitivity and specificity values equal or over 75%. It should be noted, however, that sensitivity values below this threshold may be not indicative of poor psychometric properties, as the tool may be reliable in detecting specific ASD subgroups (e.g., [41]).

## 4. Discussion

Prospective identification of early signs of ASD is widely considered a priority to ensure that children at risk of this condition have timely access to specialized services and interventions [11]. The aim of this paper was to provide healthcare and other practitioners with an overview of the technologies available to support them in the identification of overt behavioral signs of ASD in children up to six years of age. Overall, the solutions identified varied greatly in terms of screening modalities (e.g., questionnaires, behavior observations), type of interface used (e.g., tablets, eye tracker), the granularity of behavioral indicators used to estimate the risk for ASD (e.g., from subtle eye movements to behaviorally defined clinical symptoms), intended technology users (e.g., parents, clinicians), and age ranges covered by the screening tools developed. Notwithstanding such variability, psychometric information point to considering available technologies as promising support in clinical practice to detect early sign of ASD in young children. In light of these findings, some considerations may be put forward. 

First, one of the main barriers to ASD screening seems to be implementing such activity within routine clinical practice due to lack of administration or scoring time [9]. The literature identified in the current review suggests that the administration and the scoring of either existing (e.g., M-CHAT) or newly developed parent-reported questionnaires can be automated through machine learning (ML). Such ML-based solutions can be implemented within the EHR of specific primary care or specialized services (e.g., CHICA), and are effective in reducing the burden on care staff. Specifically, the evidence reviewed indicates a rapid increase in the number of children screened for ASD during the visits. Despite such encouraging results, however, it remains unclear whether clinicians would take advantage of this automated approach to screening. For instance, in the study by Downs et al. [32], almost half of positive M-CHAT results were not followed up by clinicians. A possible strategy to cope with this issue may be automating the whole screening process to ensure that at-risk children are properly assessed [32].

Second, several mobile solutions have been developed that allow data collection on children’s behaviors in non-clinical settings (e.g., home). The most affordable and effective solutions include the use of smartphones to record videos of children in their daily contexts which are subsequently analyzed (i.e., scored) by expert clinicians [37,39]. In these studies, home-made videos could be further supplemented by short questionnaires to improve the accuracy of the screening process. Alternatively, Young et al. [42] substituted text-based with video-based questionnaires to enable detection of ASD in infancy and clearly showed that video can be used to improve parent reporting of early development. Together, mobile-based solutions may be considered a strategy to (a) reduce the burden on health services, (b) increase the number of screened children, and (c) accelerate the diagnostic process. Further research is needed, however, to explore whether these mobile-based screening strategies can be effective also when used in other settings and by other users, such as kindergartens and pre-school teachers. Indeed, there are limited screening tools developed for these stakeholders (i.e., pre-school teachers), despite their importance as informants of ASD children’s social behaviors compared to their normative peer groups [63,64]. As mobile, interactive, and smart technologies (e.g., smartphones, tablets, robots) are becoming increasingly available in educational settings to foster children’s learning and creativity (e.g., [65,66,67]), teachers can be trained to use them also to contribute to the screening of young children, thus providing valuable information on children’s behavior in socially rich environments (e.g., kindergartens; primary schools). 

Third, encouraging evidence is available on the use of technology combined with ML to detect early signs of ASD through the monitoring and successive analysis of bio-behavioral markers, such as speech, movement and gaze behavior. In particular, monitoring of eye gaze behavior by means of an eye tracker resulted in the most used screening strategy to (a) distinguish between children at risk and neurotypical children (e.g., [49,51]), (b) perform L2 screening procedures (e.g., [38]), or (c) identify ASD subgroups [41]. Overall, current evidence suggests that monitoring of eye gaze should not be considered as a replacement of more traditional screening practices (e.g., parent-reported questionnaires), but an additional source of information about early signs of ASD. As already mentioned, screening is indeed widely considered a multistep process, whereby failing a L1 assessment would require a secondary screener (L2) before initiating a diagnostic process [27]. Likely, based on present findings, we argue that the increased availability of affordable and reliable eye trackers could facilitate the diffusion of this screening strategy in a variety of contexts as L2 screeners. However, more research is needed on (a) the integration of this technology in routine clinical practice, (b) whether the use of eye trackers is acceptable to clinicians, and (c) how the information gathered from the analysis of the eye movement of children can be integrated with the results obtained from more traditional screening tests. 

Voice recordings and movement observation, as well as social robots, were also further strategies identified in the present review to screen for ASD in young children (e.g., [52,53]). Although promising, however, these emerging technologies may be considered at an earlier stage of development compared to eye tracking. 

Fourth, maturity of screening solutions in terms of technological development was found to be well balanced across maturity levels (i.e., Publicly Available, Functional Prototypes), but highly unbalanced for what concerns the level of screening. Specifically, almost all the solutions included in the Publicly Available category belong to L1 (or universal) screening tools. This is not surprising given that the majority of the L1 screening solutions identified are parent-reported questionnaires which included already validated (and available) tools (e.g., M-CHAT). Based on this finding, it can be argued that the transition from traditional to technology-based screening tools may be primarily based on adaptation from currently available forms of screening strategies (i.e., questionnaires). 

Fifth, understanding the feasibility, acceptability, and effectiveness of implementing telehealth assessment is becoming of fundamental importance to cope with the limitations to health services delivery due to either low resources available (e.g., lack of trained staff), or public health emergencies (e.g., coronavirus disease 2019) [68,69]. As showed in the study by Talbott et al. [43], this approach required the active involvement of parents who had to elicit target behaviors and collect data to be shared with expert clinicians. Though telehealth assessment resulted acceptable to parents, more research is needed to understand the applicability of telehealth assessment to those parents who may experience language barriers or are less confident with technology. 

Sixth, despite we attempted to provide a comprehensive overview of the technology-based solutions available to screen for ASD, some limitations may have reduced the number of potentially relevant screening solutions. For instance, we excluded papers reporting on screening tools at a conceptual design phase that were not tested with the target population. Two further limitations include the decision (a) to focus on screening tools to assess overt children’s behaviors, thus excluding technologies to detect biological markers related to ASD condition, and (b) to exclude the literature focusing exclusively on ML-approaches to ASD screening that was not implemented in clinical settings. 

In conclusion, the results of the present review of the literature suggest that technology may be a valuable support for ASD screening. Already validated parent-reported questionnaires may be easily adapted to be administered through mobile platforms to speed up the administration and scoring processes. Commercially available mobile technologies may be used to extend the screening process to children’s life settings (e.g., home, kindergartens). In addition, more sophisticated technologies such as eye-trackers may be considered as a valid supplement to traditional screening measures.

## Figures and Tables

**Table 1:** Studies included in the review.

Study	Publication * ^1^	Participants (n); Sex (M/F); Age	Diagnostic Tools	Type of Interface ** ^1^	Technology Used	ASD Indicator Used	User	Context of Use/Implementation	Level of Screening	Maturity *** ^1^
Abbas et al. (2018) [37]	CO-J	Children referring to autism centers (n = 230); (N/A); 18–72 months	ADOS	PC, M	Parent-completed questionnaires and experts’ video tagging (*Cognoa*)	Behavioral (parent-reported; clinical observation)	Caregivers; clinicians	Community; clinic (USA)	L1	P (https://cognoa.com/)
Anzulewicz et al. (2016) [48]	SBS-J	(a) with ASD (n = 37); (24/12); 3–6 years (b) neurotypical (n = 45); (32/13); 3–6 years	Not reported	NUI	iPad-based gameplay	Behavioral (motor patterns)	Caregivers, clinicians	Laboratory (UK)	L1	F
Bauer et al. (2013) [22]	MED-J	Children visited at 18- and 24-month (n = 857) (a) with MCHAT (n = 567) (b) MCHAT high-risk (n = 171)	M-CHAT	PC	HER: Child Health Improvement Through Computer Automation system (CHICA)	Behavioral (parent-reported)	Clinicians	Clinics (USA)	L1	P (https://medicine.iu.edu/pediatrics/specialties/health-services/child-health-informatics-research-development-lab/the-chica-system)
Ben-Sasson et al. (2018) [47]	MED-J	Children with suspect of ASD (parental concerns) (n = 115) (a) with ASD-family member (n = 66); (N/A); 16–30 months (b) without ASD-family member (n = 49); (N/A); 16–30 months	M-CHAT-R/F; ASQ	PC, M	Automatic analysis of web-based discussion forums for parents with ASD concerns	Behavioral (parent-reported)	Caregivers	Community (Israel; USA)	L1	F
Campbell et al. (2017) [31]	MED-J	Children referred to a primary care visit (n = 1191) (a) Baseline period (n = 657); (321/336); M: 21.89 (3.38) months (b) Intervention period (n = 534); (275/259); M: 21.88 (3.46) months	M-CHAT-R/F	M	Digital (tablet-based) version of M-CHAT-R/F	Behavioral (parent-reported)	Caregiver	Clinic (USA)	L1	P (https://m-chat.org/)
Carpenter et al. (2020) [36]	AU-J	Children recruited at pediatric primary care visit (a) neurotypical (n = 74); (43/31); M: 21.7 (3.8) months (b) Non-ASD delay (n = 8); (5/3); M: 23.9 (3.7) months (c) ASD (n = 22); (17/5); M: 26.2 (4.1) months	M-CHAT-R/F; ADOS	M, NUI	Tablet-based facial expressions assessment	Behavioral (facial expressions)	Clinician	Clinic (USA)	L1	F
Crippa et al. (2015) [52]	AU-J	Convenience sample (n = 30) (a) neurotypical (n = 15); (12/3); M: 2.6 (5.2) (b) ASD (n = 15); (13/2); M: 3.5 (7.7) ^2^	Griffiths Mental Development Scales; ADOS	SW	Optoelectronic system coupled with passive markers attached to the participants’ hands and wrists	Behavioral (motor patterns)	Clinicians	Laboratory/Clinics (IT)	L1	F
Downs et al. (2019) [32]	MED-J	Children referring to 4 pediatric clinics (n = 274) (a) Intervention (n = 138); (84/43); 18–24 months (b) Control (n = 136); (78/58); 18–24 months	Not reported/not relevant	PC	EHR: Child Health Improvement Through Computer Automation system (CHICA)	Behavioral (parent-reported)	Clinicians, caregivers	Clinic (USA)	L1	P (https://medicine.iu.edu/pediatrics/specialties/health-services/child-health-informatics-research-development-lab/the-chica-system)
Duda et al. (2016) [33]	AU-J	Children attending a pediatrics clinic (n = 222) (a) with ASD (n = 69); (60/9); Mdn: 3.9 (3.3) (b) non-ASD (n = 153); (109/44); Mdn: 6.6 (3.9)	Bayley Scales of Infant and Toddler Development, Third Edition; DAS-II, Wechsler Intelligence Scales for Children, Fourth Edition; Vineland Adaptive Behavior Scales, Survey Interview Form; ADOS	M; PC	Mobile Autism Risk Assessment (MARA)	Behavioral (parent-reported)	Caregivers	Clinic (USA)	L1	P (https://cognoa.com/)
Egger et al. (2018) [25]	MED-J	Children from general population (n = 1756); (1211/543); M: 40.4 (16.3) months MCHAT cohort (n = 407) (a) MCHAT high score (n = 159); (124/35); M: 24.1 (4.1) months (b) MCHAT low score (n = 248); (158/89); M: 23.2 (4.4) months	M-CHAT-R/F	M, NUI	iOS-based app (*Autism & Beyond*)	Behavioral (parent-reported and face expressions analysis)	Caregivers	Community (USA)	L1	P (https://autismandbeyond.researchkit.duke.edu/ch) No longer available for download
Frazier et al. (2018) [38]	MED-J	Children referred to a tertiary-care, multi-disciplinary ASD evaluation clinic (n = 201) (a) ASD (n = 91); (75/16); 1.6–15.8 years (b) non-ASD (n = 11); (86/24); 1.8–17.6 years	ADOS-2; SRS-2; Clinical Evaluation of Language Fundamentals—Fourth Edition (or Preschool Version—Second Edition, or the Preschool Language Scales—Fifth Edition); CBCL	NUI	Eye-tacker (*SMI RED250*)	Social attention	Clinicians	Clinic (USA)	L2	F
Gong et al. (2018) [53]	CO-C	(a) with ASD (n = 18); (14/4); 2–17 years (b) at risk (n = 8); (4/4); 3 m onths-4 years (c) neurotypical (n = 9); (2/7); 3–16 years	Not reported	NUI	iOS-based app for Apple iPod Touch	Behavioral (vocalizations)	Caregivers	Home (USA)	L1	F
Harrington et al. (2013) [34]	MED-J	Children visited at a pediatric outpatient clinic (a) prospective cohort (n = 176); M: 22.1 months (b) retrospective cohort (n = 197); M: 23.1 months	M-CHAT	M	M-CHAT on the iPad	Behavioral (parent-reported)	Clinicians	Clinics (USA)	L1	P (https://m-chat.org/)
Kanne et al. (2018) [39]	AU-J	Children referring to autism centers (n = 230) (a) ASD (n = 164); (133/31); 18–72 months (b) non-ASD (n = 66); (50/16); 18–72 months	MSEL; M-CHAT-R/F; DAS-II; SCQ; SRS; CBCL	M	Smartphone-based application (*Cognoa*)	Behavioral (parent-reported; clinical observation)	Caregivers; clinicians	Community; clinic (USA)	L1	P (https://cognoa.com/)
Martineau et al. (2011) [54]	MED-J	(a) with ASD (n = 19); (16/3); M: 118 months (b) chronological age-matched controls (n = 19); (11/8); M: 116 months (range: 41 and 118 months) (c) mental age-matched controls (n = 19); (12/7); 87 months	ADI-R	NUI	Pupil-tracker (FaceLAB monitoring system)	Pupil size	Clinicians	Laboratory (FR)	L1	F
Moore et al. (2018) [40]	AU-J	Referred/self-referred children (n = 227). (a) ASD (n = 76); (70/6); 12.1–47.4 months (b) with ASD features (n = 11); (10/1); 15.8–40.7 months (c) Developmental delay (n = 56); (36/20); 12.4–46.0 months (d) Neurotypical (n = 51); (30/21); 12.9–47.5 months (e) Other (n = 22); (11/11); 13.1–47.7 months (f) Typical sibling ASD (n = 11); (4/7); 12.2–44.6	ADOS; MSEL; VABS	NUI	Eye-tracker (*Tobii T-120*)	Social attention	Clinician	Clinic (USA)	L2	F
Oller et al. (2010) [44]	SBS-J	Children from general infant population (n = 232) (a) neurotypical (n = 106); 10–48 months (b) language delayed (n = 49); 10–44 months (c) ASD (n = 77); 16–48 months	M-CHAT	NUI	LENA (Language ENvironment Analysis)	Behavioral (vocalizations)	Clinicians	Home; community (USA)	L2	P (https://www.lena.org/)
Pierce et al. (2016) [41]	MED-J	(a) with ASD (n = 115); (88/27); 12–49 months (b) with ASD symptoms (n = 20); (15/5); 11–42 months (c) with language or global DD (n = 57); (45/12); 10–46 months (d) other clinical conditions (n = 53); (26/27); 12–43 months (e) typical development (n = 64); (35/29); 12–44 months (f) ASD siblings (n = 25); (12/13); 12–31 months	ADOS; MSEL; VABS	NUI	Eye-tracker (*Tobii T-120*)	Social attention	Clinicians	Clinic (USA)	L2	F
Ramirez-Duque et al. (2020) [56]	CO-J	(a) ASD (n = 23); (N/A); M: 6.62 (2.38) years (b) Other condition (n = 15); (N/A); M: 7.75 (2.70) years	Not reported	ROB	robot (*ONO*)	Social attention	Clinicians	Laboratory (Colombia)	L2	F (https://opsoro.ugent.be/)
Schrader et al. (2020) [35]	MED-J	Children referred to a pediatric service (n = 391); (N/A); 18–24 months	Not reported	PC, M	EHR: Smart Early Screening for Autism and Communication Disorders (Smart ESAC)	Behavioral (parent-reported)	Caregivers	Clinic (USA)	L1	P (https://autismnavigator.com/autism-navigator-for-primary-care/)
Talbott et al. (2020) [43]	AU-J	Convenience sample of at risk ASD children (n = 11); (5/6); 6–12 months	AOSI; ASQ-3/ASQ-SE-2; ECI; Infant-Toddler Checklist	PC, M	Telehealth Evaluation of Development for Infants (TEDI)	Behavioral (parent-collected)	Caregivers	Home (USA)	L2	P
Tariq et al. (2018) [50]	MED-J	(a) ASD (n = 116); (78/38); M: 4.1 (2) (b) neurotypical (n = 46); (26/20) M: 2.11 (1)	ML classifier used features taken from ADI-R ADOS-2 items.	M	Video feature classifier and ML	Social attention and behavioral (expressive language, eye-contact, emotion expression, communicative engagement and echolalia)	Caregivers (they uploaded home videos) Other non-expert raters (they coded them prior to ML analysis)	Home (USA)	L1	F
Thabtah et al. (2019) [55]	CO/MED-J	Children from general population (n = 20); (N/A); 4–11 years	Not reported	M	ASDtest app	Behavioral (parent-reported)	Caregivers	Community (Multiple languages)	L1	P (https://www.asdtests.com/#home)
Vargas-Cuentas et al. (2017) [49]	SBS-J	(a) Neurotypical (n = 23);N/A; 2–6 years (b) with ASD (n = 8); N/A; 2–6 years	No formal diagnosis was available	NUI	Tablet displaying short videos and tracking eye gaze	Social attention	Caregiver	Ambulatory (Perù)	L1	F
Wan et al. (2019) [51]	AU-J	(a) ASD (n = 37); (33/4); M: 4.7 (0.7) (b) neurotypical (n = 37); (27/10); M: 4.8 (0.4)	CARS; GDS	NUI	Eye-tacker (*SMI RED250*)	Social attention	Clinicians	Clinic (China)	L1	F
Wedyan & Al-Jumaily (2016) [45]	CO-C	Convenience sample (n = 32) (a) High risk ASD (n = 17); (9/8); 12–36 months (b) Low risk ASD (n = 15); (8/7); 12–36 months	Not assessed (risk estimation based on presence of ASD sibling and/or family history of ASD)	SW	Wrist-worn sensors	Behavioral (motor patterns)	Clinicians	Laboratory (AUS)	L2	F
Wingfield et al. (2020) [46]	CO/MED-J	Convenience sample (n = 228) (a) with ASD (n = 195); (156/39); Mdn: 2.6 years (b) neurotypical (n = 33); (28/5); Mdn: 2.3 years	Not reported	M	Pictorial autism assessment schedule (PAAS)	Behavioral (parent-reported)	Clinician	Community (Sri-Lanka)	L1	F
Young et al. (2020) [42]	AU-J	(a) ASD (n = 21); (13/8); 6–18 months (b) High risk non-ASD (n = 52); (21/31); 6–18 months (c) Low risk non-ASD (n = 37); (22/15); 6–18 months	MSEL; ADOS-2	PC, M	Self-reported video-based questionnaire	Behavioral (parent-reported)	Family/caregivers	Home/Community (USA)	L2	F

Abbreviations: ADI-R, Autism Diagnostic Interview-Revised; ADOS-2, Autism Diagnostic Observation Schedule, 2nd edition; AOSI, Autism Observation Scale for Infants; ASQ, Ages and Stages Questionnaire; ASQ-SE, Ages and Stages Questionnaire: Social Emotional; CARS, Childhood Autism Rating Scale; CBCL, Child Behavior Checklist; DAS-II, Differential Ability Scales, 2nd Edition; ECI, Individual Developmental Growth Indices, Early Communication Index; GDS, Gesell Developmental Scale; KBIT-2, Kaufman Brief Intelligence Test, second edition; M-CHAT-R/F, Modified Checklist for Autism in Toddlers, Revised with Follow-Up; MSEL, Mullen Scales of Early Learning; SCQ, Social Communication Questionnaire; SRS, Social Responsiveness Scale Second Edition. ^1^ Adapted from Kientz et al. (2020). ^2^ The two groups did not differ in terms of mental age. * AU, autism-specific; CO, computing; ED, education; MED, medical; SBS, Social/Behavioral Science. Abbreviations followed by -J indicate journal papers, -C conference papers. ** PC, Personal computers and multimedia; M, Mobile applications; SII, Shared interactive interfaces; VR/AR/M, Virtual, augmented, and mixed reality; SW, Sensor-based and wearable; NUI, Natural user interfaces; ROB, Robotics. *** F, functional prototype; P, publicly available.

**Table 2:** Analysis of the studies included in the review.

Study	Screening Process Description	Screening Duration	Methodology for Screening Evaluation	Psychometric Properties *				Other Relevant Psychometric Properties
				S*e*	S*p*	PPV	NPV	
Abbas et al. (2018) [37]	System composed of (a) a short questionnaire about the child, completed by the parent, and (b) identification of specific behaviors by trained analysts after watching 2–3 short videos of the child within their natural environment that are captured by parents using a mobile device.	Not reported	Based on the responses to the questionnaire and the analysis of the videos, the authors trained two independent ML classifiers and combined their outputs into a single screening assessment	97–98% ^1^	62–64% ^1^	N/A	N/A	Performance accuracy markedly improved when combining the two classifiers into a single one.
Anzulewicz et al. (2016) [48]	Two commercially available gameplays running on iPad (mini) were used to record children’s movements while interacting with the device. Three machine learning algorithms were employed to differentiate gestures of children with ASD from those of children without ASD	Approximately 15 min	Proof-of-concept study aimed at assessing (1) whether ASD condition can be inferred from kinematic and (2) which motor features can be used to differentiate between the two groups	76–83%	67–88%	N/A	N/A	Best accuracy (AUC) was achieved using Regularized Greedy Forest approach and resulted on average of 0.93.
Bauer et al. (2013) [22]	Upon check-in to the clinic, CHICA administers two pre-screener questions for the parent to complete in the waiting room. MCHAT may be also administered (at 24-month visit only) and automatically scored. The results of the pre-screening process are provided to the clinician before the visit.	Not reported	To assess change in ASD screening rates after implementation of CHICA at two community-based clinics	N/A	N/A	N/A	N/A	Not reported
Ben-Sasson et al. (2018) [47]	System combining (a) automated text analysis relative to parental concerns with (b) minimal standard questioning taken from MCHAT-R to identify risk of ASD	Not reported	Proof-of-concept study assessing the association between the text analysis combined with standard questions and clinician’s ratings of ASD risk on a scale from 1 (no risk) to 4 (high risk).	N/A	N/A	N/A	N/A	System accuracy (AUC) range 0.74–0.88
Campbell et al. (2017) [31]	The digital M-CHAT-R/F automatically scored answers provided by parents and presented and scored follow-up questions for secondary screening of medium risk results (score of 3–7). The score report was provided to the physician before the visit.	About 20 min	Prospective study assessing the uptake of the digital MCHAT on service process measures (i.e., accuracy of documentation of screening results and appropriate action for positive screens). Acceptability was also investigated with participating physicians.	N/A	N/A	N/A	N/A	
Carpenter et al. (2020) [36]	Short movies presented on a tablet. The embedded tablet camera recorded facial movement so that affect and head position could be subsequently analyzed by means of computer vision analysis.	About 10 min	Proof-of-concept study assessing the feasibility and accuracy of the tablet-based screening procedure. Participants were recruited at their pediatric primary care visit.	N/A	N/A	N/A	N/A	System accuracy (AUC) range 75–83 (including age as covariate)
Crippa et al. (2015) [52]	Optic sensors used to track children while performing reaching, grasping, and dropping movements.	No reported	Proof of concept study to test the predictive value of the ML approach comparing the performance of neurotypical children with those of children already diagnosed as autistic.	82.2%	89.1	N/A	N/A	Overall mean classification accuracy (specificity/sensitivity) resulted 84.9 %
Downs et al. (2019) [32]	Child Health Improvement Through Computer Automation system (CHICA). Based on EHR information and pre-screen questions answered by parents, CHICA alerts the clinicians to either refer the child for an ASD evaluation or administer the M-CHAT-F (or M-CHAT-R/F).	Multi-phase process	Randomized-controlled trial involving 4 clinics (2 using CHICA with ASD module; 2 using CHICA without ASD module) to assess the percentage of children at the 18-month or 24-month visits.	N/A	N/A	N/A	N/A	None reported
Duda et al. (2016) [33]	Caregivers answered to a 7-item questionnaire presented on any digital device. Answers are automatically analyzed by a ML algorithm which classifies children (i.e., at risk; not at risk).	5–10 min	Prospective study to test the predictive values of the MARA in a clinical sample of children referred for developmental/behavioral concerns and assessed for ASD using a gold standard procedure (ADOS; Bayley; WISC; Vineland).	89.9% ^2^	79.7% ^2^	67% ^2^	95% ^2^	
Egger et al. (2018) [25]	iOS-based app running on iPhone/iPad presenting the parents (1) brief questionnaires addressing parental and child’s status (i.e., tantrums), and (2) four short movies to the child. The camera on the device records a video of the child’s face as s/he watches the movies. Caregivers upload either the whole videos of their child or only the facial landmarks. Then, emotions (positive/negative) and attention are automatically encoded.	Not reported	Exploratory study aimed at assessing acceptability and feasibility of the app. Associations of the automatically coded emotions and behaviors with age, sex, and autism risk status (MCHAT score) were assessed.	N/A	N/A	N/A	N/A	
Frazier et al. (2018) [38]	Children were shown a series of scenes representing 7 distinct stimulus paradigms (e.g., gaze following and joint attention; abstract shape movement)	5–10 min ^3^	Proof-of-concept study to validate an Autism Risk Index (ARI) and an Autism Severity Index (ASI) using eye tracking metrics.	N/A	N/A	N/A	N/A	ARI test accuracy (AUC) for children < 4years and +4 years was 0.92 and 0.93 respectively. ASI resulted strongly associated with ADOS-2 total severity scores (r = 0.58–0.67)
Gong et al. (2018) [53]	The app recorded the vocalizations of children (a) while they played gamified exercises (e.g., reading a story, describing a picture) and (b) in their life environments during everyday communications.	Whole day, multiple days	Proof-of-concept study to assess classification accuracy based on acoustic and language features of children’s vocalization over a 17 month period of use.	N/A	N/A	N/A	N/A	Classification accuracy using unweighted average F1-score was 88.9%
Harrington et al. (2013) [34]	M-CHAT on the iPad provided to children’s parents while they were being triaged.	About 2 min ^4^	To compare the effectiveness of the M-CHAT on an electronic format versus paper format in an outpatient clinic setting. Parents were also asked to rate their experience with the iPad M-CHAT. The study did not perform follow-up on the final diagnosis of patients who were screened	N/A	N/A	N/A	N/A	
Kanne et al. (2018) [39]	*Cognoa* tool includes (a) 15-item parent-report questionnaire; and (b) a 1–2 min. home video observation of the at-risk child captured via parent smartphone (see also Abbas et al., 2018).	Not reported	The performance of *Cognoa* in detecting at risk children was compared with ASD screening measures (MCHAT-R/F; SRS; SCQ; CBCL).	75% ^5^	62% ^5^	83% ^5^	50% ^5^	
Martineau et al. (2011) [54]	Participants’ eye gaze were monitored while looking at images on a computer screen to obtain a task-evoked pupil measurement and to test differences between dark and light conditions	No reported	Experimental study to test baseline pupil size and pupil responses to visual stimuli (faces, objects, and avatar) in three groups: (1) ASD; (2) age-matched; (3) mental age-matched.	N/A	N/A	N/A	N/A	Pupil size correctly predicted group membership classification in 89% of the participants in the ASD group; in 63% in the mental age-matched group, and in 63% in the chronological age-matched group
Moore et al. (2018) [40]	Replication of GeoPref Test (Pierce et al. (2016)) with the inclusion of longer and more complex social scenes.	From 60 s. (Original version) to 90 s. (Complex version)	Experimental study investigating the predictive values of two combined versions of the GeoPref test (Complex/ Original social scenes).	35% ^6^	94% ^6^	72% ^6^	78% ^6^	Classification accuracy (AUC) was 0.75
Oller et al. (2010) [44]	LENA (Language ENvironment Analysis) recording device used to acquire whole day recordings of infants in their natural environments	Whole day	Proof of concept study assessing an automated procedure (i.e., algorithm) to differentiate vocal recordings from neurotypical children, children with language delay, and those with ASD	75%	98%	N/A	N/A	The system also differentiated the neurotypical children from a combined autism and language-delay sample (S*p* = 90%).
Pierce et al. (2016) [41]	GeoPref Test consisting of 2 dynamic images presented side-by-side for a total of 60 s. One side featured a social stimulus (e.g., children dancing); the other side featured short sequences of moving geometric shapes. The side (left/right) of presentation scenes was randomly assigned	60 s	Cross-sectional explorative study investigating the relationship between percent of viewing time of geometric and social scenes and clinical measures (ADOS; Mullen Scale of Early Learning, and VABS) to assess specificity, sensitivity, and positive/negative predictive values of GeoPref Test. Test-retest was also assessed.	21% ^7^	98% ^7^	86% ^7^	70% ^7^	
Ramirez-Duque et al. (2020) [56]	Social robot (ONO) used to elicit and assess joint attention during triadic (i.e., child-therapist-robot) interactions.	Not reported	Proof of concept study assessing differences in joint attention between children with a diagnosis of ASD and children with other neurodevelopmental disorders.	N/A	N/A	N/A	N/A	
Schrader et al. (2020) [35]	Smart Early Screening for Autism and Communication Disorders (Smart ESAC). A digital tool including (a) 10-question screen for communication delay, which, if positive, is followed by (b) 20 autism-specific screening questions.	15–20 min	Pre- and post-Smart ESAC implementation data were compared to assess impact on referral and intervention timing.	81–84%	70–89%	N/A	N/A	
Talbott et al. (2020) [43]	The Telehealth Evaluation of Development for Infants (TEDI) involves parents’ delivery of semistructured parent–child play interactions using both direct coaching and written materials	Not reported	Feasibility study to assess the use of laboratory/clinical measurements (e.g., AOSI) by parents.	N/A	N/A	N/A	N/A	
Tariq et al. (2018) [50]	Families uploaded home videos on an internet server. Then independent non-expert raters tagged features of the videos. Then several ML classifiers were tested to evaluate and quantify risk for ASD.	Raters employed an average of 4 min to score videos.	Different ML classifiers were tested. The most effective one was a 5-feature logistic regression classifier (LR5). The features were expressive language, eye-contact, emotion expression, communicative engagement, and echolalia.	87.8 % (independent validation) 94.5% (study)	72.7% (independent validation) 77.4% (study)	N/A	N/A	Classification accuracy was 89% (LR5)
Thabtah et al. (2019) [55]	ASDTests is an app based on two short versions of the AQ and Q-CHAT screening methods. It targets 4 age ranges (≤36 months; 4–11 years; 12–16; ≥ 17 years). The app automatically computes the total score of the questionnaires compiled by the caregivers and—if the result is above a specified threshold—refers them a specialized assessment. It also produces a report in PDF.	Not reported	Not reported	92.8–98%	91.3–97.3%	N/A	N/A	Classification accuracy was 92.8–97.9% (Naïve Bayes—Logistic Regression).
Vargas-Cuentas et al. (2017) [49]	A tablet was used to present a 1-min video displaying a social scene with playing children and an abstract scene with moving shapes on either side of the screen. The child’s face was recorded while watching the video using the tablet’s front camera. Gaze preference was then calculated automatically.	5–10 min	Proof-of-concept study exploring (a) the performance of the automatic eye gaze detection algorithm compared to manual scoring, and (b) ASD children’s scene preferences.	N/A	N/A	N/A	N/A	The correlation between the manual and the automatic classifications for left/right gaze resulted 73.2%.
Wan et al. (2019) [51]	Children had to attend to a muted video clip of a female speaking while their gaze were tracked.	10 s	Proof-of-concept study comparing gaze fixations of ASD children with those of neurotypical peers to assess accuracy of the test to discriminate between the two groups by means of a machine learning method (support vector machine).	86.5% ^8^	83.8% ^8^	N/A	N/A	Classification accuracy was 85.1% [machine learning with support vector machine].
Wedyan & Al-Jumaily (2016) [45]	Wrist-worn sensors track the arm movement while the infant execute two motor tasks: (a) throw a ball into a tray; (b) fitting the ball into the tube.	Not reported	Explorative study aimed at investigating the overall classification accuracy of the two tasks in classifying the participants (high risk; low risk).	75%/76.4%	73.3%/73.3%	N/A	N/A	Accuracy for the two tasks (correctly classified/total sample) was 74.1%/75%
Wingfield et al. (2020) [46]	The Pictorial autism assessment schedule (PAAS) is a mobile-based application that can be administered by non-specialist healthcare workers in LMIC at home, to advise if a clinical referral is recommended. It includes 21 yes-no questions and involves ML to automatically detect the risk of ASD.	Not reported	A descriptive study reporting on the preliminary assessment of the accuracy of the PAAC involving a selected sample of children with and without ASD	88%	96%	N/A	N/A	Classification accuracy (AUC) was 0.98
Young et al. (2020) [42]	Infant Rating System for Autism (VIRSA) is a web-based application that presents pairs of videos of parents and infants playing together and requires parents to judge which video is most similar to their child.	Average of 56.49 s (SD = 11.49)	Parents completed VIRSA ratings when their child was 6-, 9-, 12-, and 18-months-old and again 2 weeks later to examine test–retest reliability.	100% ^9^	53% ^9^	0.19% ^9^	100% ^9^	(a) Split-half reliability (r = 0.48); (b) test–retest reliability (72% agreement); (c) convergent validity correlation with concurrent ADOS-2 [SARRB algorithm scores at 18 months] (r = −0.36)

Abbreviations: ADOS, Autism Diagnostic Observation Schedule; AOSI, Autism Observation Scale for Infants; EHR, Electronic Health Records; M-CHAT/M-CHAT-R/F, Modified Checklist for Autism in Toddlers, Revised with Follow-Up; ML, machine learning. * Considered as the extent to which these tests are able to identify the *likely presence or absence* of a condition of interest so that their findings encourage appropriate decision making. Appropriate psychometric information includes: Sensitivity (S*e*), Specificity (S*p*), and Predictive Values (positive [PP], and Negative [NP]). ^1^ Based on results from the parent questionnaire only (all ages). ^2^ Total sample. ^3^ Estimate based on a similar study by Frazier et al. [57]. ^4^ According to the opinion of the majority of respondents (45.1%). ^5^ Entire age range (18–72 months). ^6^ All available subjects (n = 126; 69% Geo threshold). ^7^ Using 69% Geometric Fixation Cutoff. ^8^ Fixation time for the body and mouth. ^9^ for 18-month VIRSA with concurrent 18-month diagnosis.

## Data Availability

The data presented in this study are openly available in Open Science Framework. doi: 10.17605/OSF.IO/8Y9RG. In detail, all the information concerning selection and scoring of the papers’ titles can be found here: https://mfr.osf.io/render?url=https://osf.io/udezy/?direct%26mode=render%26action=download%26mode=render. All the information concerning the selection and scoring of the papers’ abstracts can be found here: https://mfr.osf.io/render?url=https://osf.io/kx87p/?direct%26mode=render%26action=download%26mode=render.

## Appendix A

**Table A1:** PubMed search terms.

Search ID	Search Terms
1	Child, Preschool (Mesh)
2	Infant * or baby or babies or toddler * or girl * or boy * or pre * school *
3	#1 OR #2
4	Autism Spectrum Disorder (Mesh)
5	autis * or asperger * or pervasive or PDD or PDDNOS or pervasive develop * or autistic
6	#4 OR #5
7	3# AND #6
8	Technology (Mesh)
9	Computer or mobile or digital or smart or wearable * or ICT or information technology or electronic or device or smartphone or mobile phone or virtual reality or robots or social robot * or augmented reality or speech generating device or SGD or iPad or tablet or eye tracker or gaze tracker or eye tracking or sensors or artificial intelligence or AI or voice-controlled or personal assistants or virtual assistants or smartwatch or iWatch or smartglasses or GPS or assistive technology or AT or internet of things or IOT
10	#8 OR #9
11	Early Diagnosis (Mesh)
12	Early Medical Intervention (Mesh)
13	Early Intervention, Educational (Mesh)
14	#11 OR #12 OR #13
15	#10 AND #14
16	#7 AND #15

**Table A2:** EBSCO and Web of Science (WoS) search terms.

Search ID	Search Terms
1	Infant * or baby or babies or toddler * or girl * or boy * or pre * school *
2	autis * or asperger * or pervasive or PDD or PDDNOS or pervasive develop * or autistic
3	#1 AND #2
4	Technology or Computer or mobile or digital or smart or wearable * or ICT or information technology or electronic or device or smartphone or mobile phone or virtual reality or robots or social robot * or augmented reality or speech generating device or SGD or iPad or tablet or eye tracker or gaze tracker or eye tracking or sensor * or artificial intelligence or AI or voice-controlled or personal assistants or virtual assistants or smartwatch or iWatch or smartglasses or GPS or assistive technology or AT or internet of things or IOT
5	Diagnosis or Screening or Early Intervention or Preschool Education
6	#4 AND #5
7	#3 AND #6

* Limiters—Published Date: 19900101-20191231; Expanders—Apply equivalent subjects; Narrow by Language—English; Narrow by SubjectAge:—preschool age (2–5 yearrs); Narrow by SubjectAge—childhood (birth-12 yearrs); Search modes—Boolean/Phrase.

**Table A3:** Institute of Electrical and Electronics Engineers (IEEE) search terms.

Search ID	Search Terms
1	preschool
2	Infant *
3	#1 OR #2
4	Autism Spectrum Disorder (Mesh)
5	autis* or pervasive
6	#4 OR #5
7	3# AND #6

**Table A4:** Association for Computing Machinery (ACM) search terms.

Search ID	Search Terms
1	(All: autism) AND (All: infant)

## Appendix B

In this first step, the titles of the papers retrieved will be reviewed by three independent researchers (Lorenzo Desideri, Patricia Pérez-Fuster, and Gerardo Herrera) and scored as not relevant (0), probably relevant (1), or relevant (2). The scores will be added to make a sum score ranging from 0 to 6. All publications with a sum score of 6 will be selected for the next step. In general, in case of doubt please keep the tittle in the list (i.e., if the age-range is not specified, if the target population is not clear or if it may include ASD together with other populations, or if it is not clear whether it is related to screening/monitor/intervention or not, or if it is not clear if it is a review paper or a primary study).

**Table A5:** Instructions for titles scoring.

Score	Instructions	Examples
0 points	(a) Title refers to a different age range than 0–6 OR (b) Title refers to a different term than autism (i.e., elderly or cerebral palsy, but not autism related terms) OR (c) Title refers to a different application area than Screening/ Monitoring or Intervention OR (d) Title is related to a systematic review or meta-analysis (instead of being a primary study) OR (e) Tittle is related to genetic/biochemical research	Title 1: “Digital images as meaning bridges: Case study of assimilation using avatar software in counselling with a 14-year-old boy” Explanation: The study satisfies two inclusion criteria: (1) it involves autism, (2) it refers to a technology-based intervention. However, it is explicitly mentioned that it does not focus on pre-school children. Title 2: “Technology-mediated learning in students with ASD. A bibliographical review” Explanation: The study satisfies two inclusion criteria: (a) it involves autism, (b) it refers to a technology-based intervention. However, it is a systematic review.
1 point	(a) Title includes any term related to autism spectrum disorder condition (autis* or Asperger* or pervasive or PDD or PDDNOS or pervasive develop* or autistic) OR (b) Title refers to (any kind of) technology-based intervention or screening (or monitoring) AND (c) Title does not qualify for any of the 5 options that apply for 0 points	Title 1: “Sustained Community Implementation of JASPER Intervention with Toddlers with Autism” Explanation: The article refers to autism (and toddlers) which is the focus of our study. Even if we don’t know whether JASPER is a technology-based intervention, it is worth including this article in the next step. Title 2: “Factor Analysis of the Childhood Autism Rating Scale in a Sample of Two Year Olds with an Autism Spectrum Disorder” Explanation: The study satisfies two inclusion criteria: (a) it involves autism, (b) it refers to a tool for diagnosis. I know that the Childhood Autism Rating Scale is an observational tool, but I would prefer to be highly inclusive in this very first step.
2 point	(a) Title includes any term related to autism spectrum disorder condition (autis* or as-perger* or pervasive or PDD or PDDNOS or pervasive develop* or autistic) AND (b) Title refers to (any kind of) technology-based intervention or screening (or monitoring) AND (c) Title does not qualify for any of the 5 options than apply for 0 points	Title 1: “Randomised controlled trial of an iPad based early intervention for autism: TOBY playpad study protocol” Explanation: The study satisfies inclusion criteria: (a) it involves autism AND (b) it refers to a technology-based intervention. Title 2: “Automatic newborn cry analysis: a non-invasive tool to help autism early diagnosis” Explanation: The article refers to autism (and newborns). We might suppose that the mentioned “tool” is a kind of digital technology. Hence, it would be better to include this title in the next step.
